# Vitamins in the Management of Inferior Alveolar Nerve Sensory Disturbances: A Scoping Review of Randomized Studies

**DOI:** 10.3390/jcm15197375

**Published:** 2026-09-23

**Authors:** Wojciech Macek, Maciej Chęciński, Kamila Chęcińska, Maja Kosińska, Zuzanna Olejarczyk, Maciej Sikora

**Affiliations:** 1 Department of Oral Surgery, Preventive Medicine Center, Komorowskiego 12, 30-106 Cracow, Poland; ld.wojciech.macek@wp.pl (W.M.); majaakosinska@gmail.com (M.K.); 2 National Medical Institute of the Ministry of the Interior and Administration, Wołoska 137, 02-507 Warsaw, Poland; maciej.checinski@pimmswia.gov.pl (M.C.); maciej.sikora@pimmswia.gov.pl (M.S.); 3 Department of Maxillofacial Surgery, Hospital of the Ministry of the Interior and Administration, Wojska Polskiego 51, 25-375 Kielce, Poland; 4 Independent Researcher, 30-381 Cracow, Poland; zuzanna.m.olejarczyk@wp.pl; 5 Department of Biochemistry and Medical Chemistry, Pomeranian Medical University, Powstańców Wielkopolskich 72, 70-111 Szczecin, Poland

**Keywords:** mandibular nerve, mandibular nerve injuries, nerve regeneration, paresthesia, pyridoxine, scoping review, somatosensory disorders, thiamine, vitamin B12, vitamin B complex

## Abstract

**Background:** Sensory disturbances of the inferior alveolar nerve (IAN) may follow dental and maxillofacial procedures and impair oral function and quality of life. This scoping review mapped randomized evidence on vitamin-based treatment of established IAN sensory disturbances. **Methods**: The review protocol was developed prospectively and registered on the Open Science Framework (OSF; osf.io/bvd54). PubMed, CENTRAL, BASE, SciELO, Europe PMC, and the Virtual Health Library (BVS) Regional Portal were searched from inception, with searches conducted between 31 August and 19 September 2026; the LILACS collection was also searched separately within BVS. Eligible studies compared a vitamin-based intervention with placebo, no vitamin supplementation, or the same standard care without another active treatment. **Results**: Three randomized studies involving 164 analyzed participants were included. They evaluated heterogeneous B-complex regimens after implant placement, bilateral sagittal split osteotomy, or mandibular third-molar extraction. One study found no significant between-group differences in paresthesia or pain. Another reported greater improvement in sensory-disturbance-related quality of life at 3 and 6 months in a 19-participant subset. The third reported more frequent regression of sensory disturbances in the vitamin group, although no between-group significance test was provided for this comparison and key *p* values represented within-group changes. Adverse events were not systematically reported. **Conclusions**: The evidence is sparse and methodologically heterogeneous and does not support firm conclusions regarding clinical effectiveness. Better reported randomized trials using standardized sensory outcomes and explicit safety monitoring are needed.

## 1. Introduction

The inferior alveolar nerve (IAN) is susceptible to injury during dental and oral and maxillofacial procedures, including mandibular third-molar extraction, implant placement, and orthognathic surgery [1,2,3]. Such injuries may cause paresthesia, hypoesthesia, numbness, altered pain perception, or neuropathic pain, affecting oral function and patients’ quality of life [2,3]. The risk of sensory disturbances depends on the clinical setting and anatomical relationships. In implant surgery, a recent systematic review identified an association between implant proximity to the mandibular canal and the occurrence of neurosensory alterations [4].

IAN injury may involve direct mechanical trauma, including laceration, crushing, or stretching, as well as indirect compression associated with postoperative hematoma or edema. These mechanisms can impair neural function without complete nerve transection, and the severity and extent of damage influence the potential for recovery [5]. Local anesthetic injections introduce additional possible mechanisms, including needle trauma and chemical neurotoxicity [6]. Articaine has received particular attention because some retrospective reports have associated its use, especially in 4% solutions, with persistent sensory disturbances. However, these observations do not establish causation, and randomized clinical evidence has not demonstrated a higher risk of major adverse effects than with lidocaine. Uncertainty remains regarding rare persistent nerve injuries [6,7].

Clinical assessment must capture both sensory impairment and its consequences for the patient. A prospective study of post-traumatic trigeminal neuropathy found that relationships between neurosensory findings and quality of life differed across sensory measures, supporting the complementary use of clinical testing and patient-reported outcomes [8]. Contemporary approaches emphasize a detailed history, neurosensory examination, appropriate imaging, and follow-up to inform observation, symptom management, or specialist referral [9,10]. Nevertheless, the evidence supporting individual treatments remains limited. An international expert consensus identified greater uncertainty regarding treatment than diagnosis, while a systematic review of low-level laser therapy and microsurgery highlighted heterogeneity in the available intervention studies [9,11].

B vitamins have attracted interest because of their roles in peripheral nerve metabolism and maintenance. Thiamine (vitamin B1) contributes to cellular energy metabolism, pyridoxine (vitamin B6) participates in amino-acid metabolism and neurotransmitter synthesis, and cobalamin (vitamin B12) supports one-carbon metabolism and myelin maintenance [12,13]. These complementary functions provide a biological rationale for investigating combined preparations. However, experimental evidence of neuroprotective or regenerative effects does not establish clinical effectiveness after IAN injury, and findings from other peripheral neuropathies cannot be assumed to apply directly to focal iatrogenic lesions [13,14]. Safety also requires consideration: excessive vitamin B6 exposure can cause peripheral neuropathy, making dose and treatment duration relevant when evaluating vitamin-containing interventions [15].

A recent scoping review examined pharmacological approaches to IAN sensory disturbances, including vitamin-containing regimens, within a broader range of drug therapies [16]. A focused examination of randomized vitamin studies can complement this work by detailing the preparations, comparators, clinical contexts, sensory assessments, and reported outcomes. This distinction is particularly relevant when separating the treatment of established sensory disturbances from interventions intended to prevent their occurrence.

Given the anticipated scarcity and heterogeneity of the randomized literature, a scoping review design was selected to map the available evidence, study characteristics, and research gaps rather than to estimate a pooled treatment effect.

The aim of this scoping review was to identify, characterize, and map the available evidence from randomized studies investigating vitamin-based interventions for the treatment of sensory disturbances associated with the inferior alveolar nerve. The review was guided by the following research question: what randomized evidence is available regarding the use of vitamin-based interventions for the management of inferior alveolar nerve sensory disturbances, and what clinical effects have been reported? The review also aimed to characterize the investigated vitamin interventions, comparators, patient populations, outcome assessment methods, and reported clinical outcomes, and to identify gaps requiring further research.

## 2. Materials and Methods

### 2.1. Protocol and Registration

The protocol for this scoping review was prospectively registered on the Open Science Framework (OSF; osf.io/bvd54) on 31 August 2026. The review was conducted and reported in accordance with the Preferred Reporting Items for Systematic Reviews and Meta-Analyses extension for Scoping Reviews (PRISMA-ScR) guidelines [17]. A completed PRISMA-ScR checklist is provided in Supplementary Materials S1 [18].

During revision, the information-source coverage was extended to Europe PMC and the Virtual Health Library (BVS) Regional Portal, including a separate search of the LILACS collection. This extension did not alter the eligibility criteria or the planned approach to evidence synthesis.

### 2.2. Eligibility and Exclusion Criteria

The eligibility criteria were defined a priori according to the Population–Concept–Context (PCC) framework recommended for scoping reviews [17,19].

**Population**: Studies involving human participants with sensory disturbances associated with the inferior alveolar nerve were considered eligible. Sensory disturbances included paresthesia (paraesthesia), hypoesthesia (hypesthesia), and numbness. Other eligible manifestations included reduced or absent pain sensation (hypoalgesia or analgesia), increased pain sensitivity (hyperalgesia), and impaired light-touch sensation, two-point discrimination, or thermal sensation attributable to inferior alveolar nerve injury or dysfunction.

**Concept**: The concept of interest was the use of vitamins or vitamin-containing interventions for the treatment or management of inferior alveolar nerve sensory disturbances. Eligible interventions included individual vitamins, vitamin combinations, and preparations containing vitamins, with particular consideration of vitamin B1 (thiamine), vitamin B6 (pyridoxine), vitamin B12 (cobalamin), benfotiamine, mecobalamin, methylcobalamin, cyanocobalamin, and hydroxocobalamin. Studies were eligible regardless of the dose, route of administration, treatment duration, or combination with other therapeutic modalities, provided that the contribution or use of the vitamin-containing intervention was relevant to the management of the sensory disturbance.

Eligible studies were required to compare a vitamin-based intervention with placebo, no vitamin supplementation or no treatment, or the same background standard therapy without an additional active treatment.

**Context**: No restrictions were imposed regarding the clinical setting, geographic location, or healthcare environment. Studies conducted in dental, oral and maxillofacial surgery, neurology, or other relevant clinical settings were considered eligible. Sensory disturbances could have resulted from any clinically relevant cause affecting the inferior alveolar nerve, including, but not limited to, dental procedures, oral and maxillofacial surgical procedures, trauma, or other nerve injuries.

Regarding study design, the review primarily sought to identify randomized studies evaluating vitamin-based interventions. Randomized controlled trials and other randomized comparative studies were considered eligible.

Studies were required to report at least one outcome relevant to sensory disturbance or nerve recovery, such as changes in sensory symptoms, objective sensory testing, neurological recovery, or patient-reported sensory function. No restrictions were applied regarding publication date or language.

**Exclusion criteria**: Studies were excluded if they: (1) concerned sensory disturbances affecting nerves other than the IAN and did not provide separately assessable IAN data; (2) evaluated vitamins exclusively for nutritional supplementation or prevention of nerve injury rather than for the treatment of an established sensory disturbance; (3) used the vitamin-containing intervention solely as an active comparator to another therapeutic modality; (4) were non-randomized or did not report an outcome relevant to sensory disturbance or nerve recovery; or (5) were non-original publications, including reviews, meta-analyses, protocols, editorials, commentaries, and letters without original data. Animal, in vitro, and cadaveric studies were also excluded. Duplicate publications were excluded, with the most complete or informative report retained.

### 2.3. Information Sources

A comprehensive literature search was conducted in PubMed, the Cochrane Central Register of Controlled Trials (CENTRAL), BASE (Bielefeld Academic Search Engine), SciELO, Europe PMC, and the Virtual Health Library (BVS) Regional Portal. The LILACS collection was additionally searched separately within BVS. All sources were searched from inception, without restrictions regarding publication date or language. The searches were conducted between 31 August and 19 September 2026. Europe PMC and BVS/LILACS were added during revision to broaden the coverage of the search.

### 2.4. Search

The search strategy was developed to identify randomized studies investigating the use of vitamins in patients with sensory disturbances associated with the inferior alveolar nerve. The search combined four main concepts: (1) the inferior alveolar nerve, (2) sensory disturbances or nerve injury, (3) vitamins and specific vitamin compounds, and (4) randomized study designs.

The following search strategy was used:

(“inferior alveolar nerve” OR “inferior dental nerve” OR IAN) AND (paresthesia OR paraesthesia OR hypoesthesia OR hypesthesia OR “sensory disturbance” OR “nerve injury”) AND (vitamin* OR “B1” OR “B6” OR “B12” OR cobalamin OR mecobalamin OR methylcobalamin OR cyanocobalamin OR hydroxocobalamin OR thiamine OR pyridoxine OR benfotiamine) AND (randomized OR randomised OR placebo).

The search strategy was adapted to the syntax and search functions of each source. In Europe PMC, the terms were applied to the title and abstract fields and to the keyword field. No search-engine filters regarding language or publication date were applied. The search strategy was designed to maximize sensitivity, while the predefined eligibility criteria were applied during study selection.

### 2.5. Selection of Sources of Evidence

Records retrieved from the electronic database searches were screened manually by two authors in two sequential stages: title and abstract screening, followed by full-text assessment of potentially eligible records. Duplicate records were identified and removed during the full-text assessment stage. Both authors independently assessed the records against the predefined eligibility criteria. Any disagreements regarding eligibility were resolved through discussion and consensus.

### 2.6. Data Charting Process

Data from the included studies were independently extracted by two authors using standardized data extraction tables developed for this review. The extracted information was subsequently compared between the authors. Any discrepancies were discussed and resolved by consensus. Where relevant information was unclear or incompletely reported, the corresponding study report was re-examined to ensure accurate data charting.

### 2.7. Data Items

The following data items were charted from each included study: first author and year of publication; country in which the study was conducted; study design; sample size and participant characteristics; etiology and characteristics of the inferior alveolar nerve sensory disturbance; criteria or methods used to diagnose or assess the sensory disturbance; characteristics of the vitamin intervention, including the vitamin(s) administered, formulation, dose, route of administration, treatment frequency, and duration; comparator intervention; timing of outcome assessment; sensory and neurological outcomes; methods used for outcome measurement; main findings regarding sensory recovery or improvement; and reported adverse events or treatment-related complications.

### 2.8. Synthesis of Results

The findings were synthesized descriptively and presented in tabular and narrative form. The included studies were grouped according to study characteristics, vitamin-based interventions, comparators, outcome assessment methods, and reported clinical effects. Particular attention was paid to the types and regimens of vitamin interventions, the characteristics of the treated sensory disturbances, and the range of methods used to evaluate recovery. Due to the scoping nature of the review and the expected heterogeneity of the included studies, no quantitative meta-analysis was planned. The synthesis was intended to map the available randomized evidence, identify recurring patterns in the reported findings, and highlight gaps requiring further investigation.

## 3. Results

### 3.1. Selection of Sources of Evidence

The database searches identified 44 records: 15 from BASE, 8 from the Cochrane Central Register of Controlled Trials (CENTRAL), 7 from PubMed, 6 from Europe PMC, and 8 from the BVS Regional Portal. No records were retrieved from SciELO or from the separate search of the LILACS collection. All 44 records underwent title and abstract screening, after which 9 reports were selected for full-text assessment. At the full-text stage, 5 duplicate reports and 1 ClinicalTrials.gov study protocol were excluded. The remaining 3 studies met the predefined eligibility criteria and were included in the scoping review. No additional eligible studies were identified through the searches conducted during revision. Complete agreement was achieved between the two reviewers at both the title and abstract screening stage and the full-text assessment stage. The study selection process is presented in Figure 1.

### 3.2. Characteristics of Sources of Evidence

Three randomized studies met the predefined eligibility criteria and were included in the scoping review. The main characteristics of the included sources of evidence are summarized in Table 1.

### 3.3. Results of Individual Sources of Evidence

The characteristics of the vitamin-based interventions and comparators varied across the included studies, as did the timing and methods used to assess sensory outcomes. Detailed information on the interventions, outcome measures, main findings, and reported adverse events is presented for each included study in Table 2.

### 3.4. Synthesis of Results

A summary of the mapped evidence and the main evidence gaps is provided in Table 3.

## 4. Discussion

Three randomized studies investigated vitamin B-based interventions for sensory disturbances of the inferior alveolar nerve (IAN) [20,21,22]. The studies involved different clinical etiologies, including IAN injury following dental implant placement, bilateral sagittal split osteotomy, and mandibular third-molar extraction. The interventions consisted of different B-complex formulations and varied in vitamin composition, dosing regimen, and treatment duration.

The methods used to evaluate treatment effects were heterogeneous and included clinical neurosensory examination, pinprick testing, the Global Sensitivity Score, quantitative sensory testing, VAS for pain, and patient-reported quality-of-life measures. Overall, the findings were inconsistent. Ghasemi et al. [20] observed improvement in paresthesia and pain over time but no significant differences between the vitamin B-complex and control groups. In contrast, Kamolratanakul et al. [21] reported greater improvement in sensory-disturbance-related quality of life at 3 and 6 months with vitamin B1-6-12, while Maximova and Suanova [22] reported greater regression of sensory disturbances and pain in patients receiving B-complex treatment.

Taken together, the available evidence suggests potential benefits of B-vitamin supplementation for selected outcomes; however, the small number of studies, limited sample sizes, heterogeneous interventions and outcome measures, and variable follow-up preclude consistent conclusions regarding sensory recovery. Safety data were also limited, with adverse events not reported in two of the three studies.

The available randomized evidence comprised only three studies with relatively small sample sizes, limiting confidence in the reported findings. The studies differed in the etiology of IAN injury, vitamin formulations and dosing regimens, treatment duration, follow-up periods, and methods used to assess sensory recovery. This heterogeneity limited direct comparisons and the generalizability of individual findings and precluded a meaningful quantitative synthesis, as pooling the results would not have produced an interpretable summary estimate.

The findings were therefore synthesized descriptively. Accordingly, this review maps the available randomized evidence but cannot establish the overall effectiveness of vitamin-based treatment or identify a preferred formulation, dose, or treatment duration. Safety data were limited because none of the three studies systematically reported adverse events. The absence of reported adverse events should not be interpreted as evidence that the investigated regimens were safe.

Although the search covered six electronic information sources, included a separate search of the LILACS collection, and imposed no language or publication-date restrictions, potentially relevant studies available only as unpublished or otherwise difficult-to-retrieve grey literature may have been missed. Considerable overlap between the searched sources was observed, as reflected by the duplicate reports identified during full-text assessment. Furthermore, by design, the review was restricted to randomized studies and therefore did not capture evidence from non-randomized study designs.

The differences observed across the included studies should be interpreted in the context of substantial clinical and methodological heterogeneity. Although all three studies investigated vitamin B-based interventions, the clinical circumstances in which treatment was administered differed considerably. Sensory disturbances followed dental implant placement, bilateral sagittal split osteotomy, or mandibular third-molar extraction, representing distinct mechanisms and potentially different severities of IAN injury. Consequently, the natural course of recovery and the potential responsiveness to conservative treatment may not be directly comparable across these populations.

The vitamin regimens also differed markedly. The investigated preparations contained different combinations and doses of B vitamins, and treatment duration ranged from approximately one month to several months. These differences are particularly relevant because the included studies cannot be regarded as evaluations of a single standardized therapeutic intervention. Rather, they represent a heterogeneous group of vitamin B-based strategies. Therefore, positive findings observed with one formulation or regimen should not automatically be extrapolated to other B-complex preparations.

Differences in outcome assessment may additionally explain part of the inconsistency between studies. Ghasemi et al. [20] primarily evaluated changes in paresthesia and pain, whereas Kamolratanakul et al. [21] incorporated both neurosensory testing and a condition-specific patient-reported quality-of-life measure. Maximova and Suanova [22] used clinical neurological assessment together with quantitative sensory testing and pain evaluation. These approaches capture different dimensions of sensory recovery. Improvement in a patient-reported outcome, for example, does not necessarily indicate normalization of objective sensory function, while changes detected by quantitative sensory testing may not always correspond directly to patients’ subjective perception of recovery. The lack of a standardized outcome framework therefore limits comparisons across studies and may contribute to apparently conflicting findings.

## 5. Conclusions

The randomized evidence on vitamin-based interventions for IAN sensory disturbances is limited and heterogeneous. One study reported greater improvement in sensory-disturbance-related quality of life, one reported favorable descriptive and within-group changes without a reported between-group significance test for the main clinical comparison, and one found no significant between-group differences in paresthesia or pain. Differences in populations, vitamin regimens, outcomes, and follow-up prevent firm conclusions regarding effectiveness. Better reported randomized trials using standardized sensory measures and systematic adverse-event monitoring are needed.

## Figures and Tables

**Figure 1:**
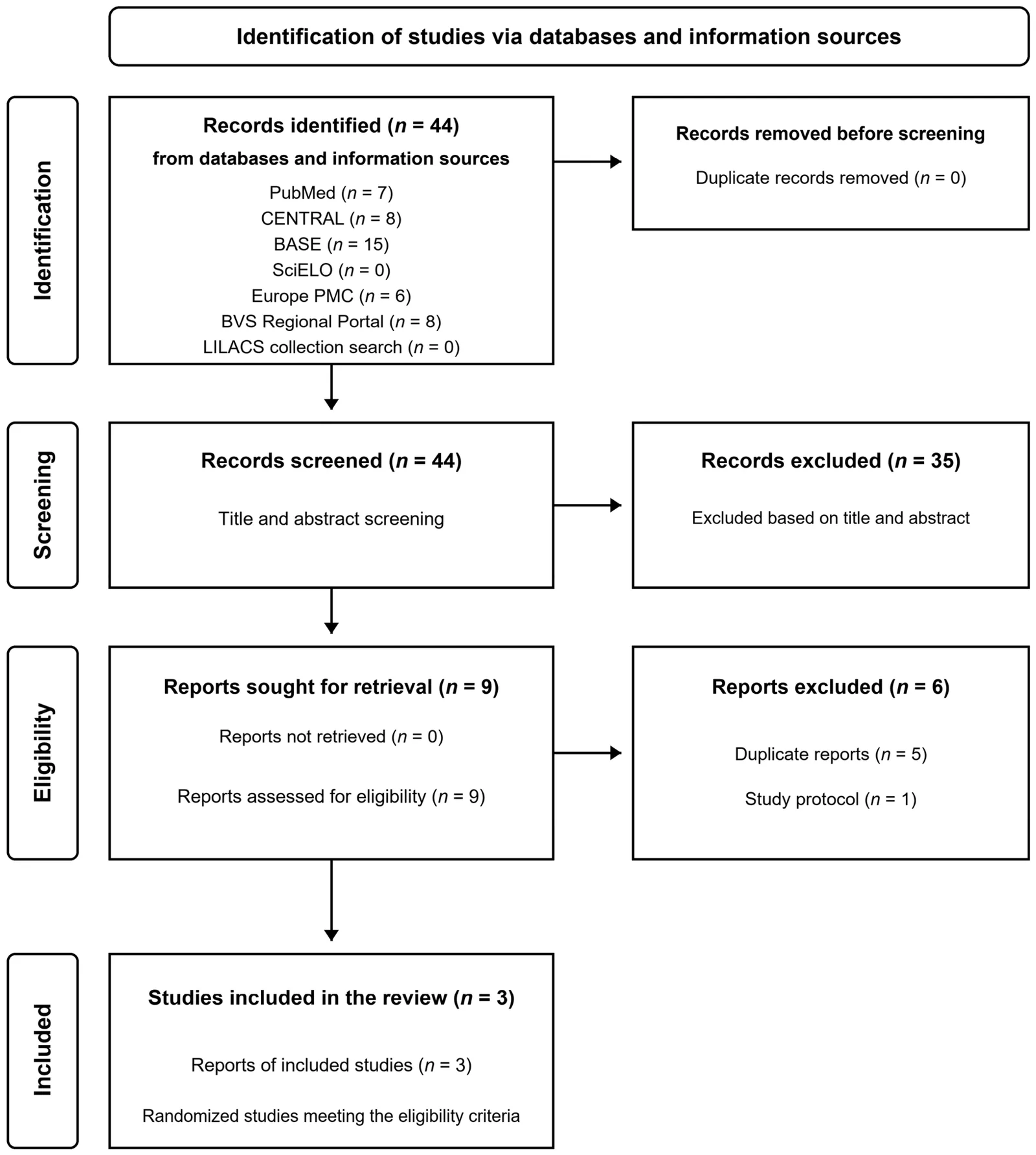
Flow diagram of the study selection process.

**Table 1:** Characteristics of the included sources of evidence.

Data Item	Study 1	Study 2	Study 3
Characteristic	Ghasemi et al., 2022 [20]	Kamolratanakul et al., 2018 [21]	Maximova & Suanova, 2024 [22]
Country	Iran	Thailand	Russia
Study design	Single-blind randomized clinical trial	Prospective, randomized, double-blind trial	Open-label randomized comparative trial
Sample size	46 patients (23 intervention; 23 control)	32 patients (18 vitamin B1-6-12; 14 control)	86 patients (44 intervention; 42 control)
Participant characteristics	Healthy patients requiring dental implants in the posterior mandibular region; patients with altered lower-lip sensation due to previous mandibular surgery or third-molar extraction were excluded	Healthy patients aged 18–40 years who underwent mandibular orthognathic surgery with BSSO and developed postoperative sensory impairment (Global Sensitivity Score < 12)	Patients with traumatic IAN injury and facial sensory disturbances persisting for 72 h after mandibular third-molar extraction; age reported as 40–59 years in Methods and 45–54 years in the abstract.
Etiology of sensory disturbance	IAN injury following mandibular dental implant placement	IAN injury following mandibular orthognathic surgery with bilateral sagittal split osteotomy (BSSO)	Traumatic IAN injury following surgical extraction of a mandibular third molar
Characteristics of inferior alveolar nerve sensory disturbance	Paresthesia; severity assessed by pinprick test; pain was also evaluated	Postoperative sensory impairment/IAN dysfunction defined by Global Sensitivity Score < 12	Numbness involving the teeth, lower lip and jaw; hypoalgesia, hypoalgesia with areas of analgesia, or hyperalgesia; constant drilling pain of varying intensity
Diagnostic/assessment criteria	IAN injury assessed within 24 h after implant placement according to the IANIDIS protocol and by the project supervisor; severity recorded using the pinprick test; subsequent changes in sensation assessed by standard neurosensory examination; pain assessed using a 10-cm VAS	Global Sensitivity Score based on static two-point discrimination, moving two-point discrimination, light-touch sensation and pinprick sensation; score < 12 defined sensory impairment. CS-OIDP was additionally used to assess quality-of-life impact specifically related to neurosensory disturbance	Standard clinical and neurological examination; 10-cm VAS for orofacial pain; standardized quantitative sensory testing (QST) using TSA II with assessment of thermal and pain thresholds; thermal thresholds compared with DFNS reference values

Abbreviations: IAN, inferior alveolar nerve; IANIDIS, Inferior Alveolar Nerve Injury Diagnostic and Information System; VAS, visual analogue scale; GSS, Global Sensitivity Score; CS-OIDP, Condition-Specific Oral Impact on Daily Performance; QST, quantitative sensory testing; TSA II, Thermal Sensory Analyzer; DFNS, German Research Network on Neuropathic Pain.

**Table 2:** Results of individual included studies.

Data Item	Ghasemi et al., 2022 [20]	Kamolratanakul et al., 2018 [21]	Maximova & Suanova, 2024 [22]
Vitamin intervention	Vitamin B complex as an adjunct to routine post-implantation treatment	Vitamin B1-6-12 supplementation	B-complex preparation (Combilipen) in addition to standard therapy
Vitamin(s) administered	Vitamin B1, B2, B6 and nicotinamide	Vitamin B1, B6 and B12	B-complex preparation; individual vitamin components NR
Formulation	Tablets	NR	Tablets
Dose	B1 5 mg, B2 2 mg, B6 2 mg, nicotinamide 20 mg per tablet	B1 300 mg, B6 15 mg, B12 165 μg *	1 tablet per administration; vitamin-specific dose NR
Route of administration	Oral	Oral	Oral
Treatment frequency	Daily	Three times daily	1 tablet three times daily
Treatment duration	From immediately after implantation; exact total duration NR	Until sensation returned to normal or up to 6 months after surgery	30 days
Comparator	Routine treatment after implantation + placebo	No vitamin B prescription	Standard therapy only: chlorhexidine mouth rinses
Outcome assessment time points	14 days, 1 month, 2 months and 3 months after treatment	1 week, 1 month, 3 months and 6 months after surgery	Before treatment and after the 30-day treatment course
Sensory/neurological outcomes	Paresthesia/sensory changes; pain intensity	Sensory impairment related to IAN injury; impact of neurosensory disturbance on quality of life	Sensory disturbances including hypoalgesia, hypoalgesia with areas of analgesia, hyperalgesia and numbness; pain; thermal and pain sensitivity thresholds
Outcome measurement methods	Pinprick test for initial severity; standard neurosensory examination for changes in sensation; 10-cm VAS for pain	Global Sensitivity Score based on static two-point discrimination, moving two-point discrimination, light-touch sensation and pinprick sensation; OIDP and CS-OIDP for sensory-disturbance-related quality of life	Standard clinical and neurological examination; 10-cm VAS; quantitative sensory testing (QST) using TSA II for cold/warm sensation and cold/heat pain thresholds
Main findings	Pain intensity decreased over 3 months in both groups, but no significant differences between intervention and control at any follow-up. Paresthesia also decreased in both groups, with no significant between-group differences at any follow-up.	Among 19 of the 32 participants whose quality-of-life impact was attributed primarily to sensory impairment, CS-OIDP improvement was greater in the vitamin B1-6-12 group at 3 months (82.38% vs. 35.33%, *p* = 0.03) and 6 months (86.5% vs. 3.45%, *p* = 0.003); no significant difference was found at 1 month.	Regression of sensory disturbances was reported in 40/44 (90.9%) patients in the B-complex group and 20/42 (47.6%) controls; no between-group significance test was reported for this comparison. VAS pain decreased from 6.3 ± 0.9 to 0.8 ± 0.2 in the intervention group (within-group *p* = 0.02) and from 6.1 ± 0.6 to 4.8 ± 0.4 in controls (within-group *p* = 0.09). The warm-sensation threshold decreased within the intervention group (36.54 ± 0.88 to 33.78 ± 0.92 °C; *p* = 0.03), but no significant between-group difference after treatment was reported.
Adverse events/complications	NR	NR	NR

Abbreviations: NR, not reported; VAS, visual analogue scale; GSS, Global Sensitivity Score; OIDP, Oral Impacts on Daily Performances; CS-OIDP, Condition-Specific Oral Impact on Daily Performance; QST, quantitative sensory testing; TSA II, Thermal Sensory Analyzer. * The source report contains inconsistent dosing descriptions: the abstract presents vitamin B1 300 mg, vitamin B6 15 mg, and vitamin B12 165 μg as daily doses, whereas the Methods Section states that these amounts were administered three times daily.

**Table 3:** Synthesis of evidence from randomized studies investigating vitamin-based interventions for inferior alveolar nerve sensory disturbances.

Data Item	Summary of Findings
Vitamin interventions investigated	B-complex preparations containing different combinations of vitamins B1, B2, B6, B12, and nicotinamide.
Number of studies	3
Patient populations and etiologies	Patients with IAN sensory disturbances following dental implant placement, mandibular orthognathic surgery, or mandibular third-molar extraction.
Comparators	Routine postoperative treatment plus placebo, no vitamin B supplementation, or standard therapy alone.
Outcome assessment methods	Neurosensory examination, pinprick testing, Global Sensitivity Score, quantitative sensory testing (QST), VAS for pain, and CS-OIDP.
Overall findings regarding sensory recovery	Two studies reported benefits of B-vitamin treatment for selected sensory-related outcomes, whereas one found no significant between-group difference in paresthesia despite improvement over time in both groups.
Consistency of findings across studies	Findings were not fully consistent, and studies differed in injury etiology, vitamin regimen, treatment duration, follow-up, and outcome assessment.
Reported adverse events	Adverse events were not systematically reported in any of the included studies.
Key evidence gaps	Limited number and size of studies, heterogeneous interventions and assessment methods, variable follow-up, and limited safety data.

Abbreviations: IAN, inferior alveolar nerve; QST, quantitative sensory testing; VAS, visual analogue scale; CS-OIDP, Condition-Specific Oral Impact on Daily Performance; B1, vitamin B1 (thiamine); B2, vitamin B2 (riboflavin); B6, vitamin B6 (pyridoxine); B12, vitamin B12 (cobalamin).

## Data Availability

All data analyzed in this study are presented in the article.

## Supplementary Materials

The following supporting information can be downloaded at: https://www.mdpi.com/article/10.3390/jcm15197375/s1, Material S1: PRISMA 2020 Checklist.

## Abbreviations

The following abbreviations are used in this manuscript:

BSSO	Bilateral sagittal split osteotomy
CS-OIDP	Condition-Specific Oral Impact on Daily Performance
GSS	Global Sensitivity Score
IAN	Inferior alveolar nerve
IANIDIS	Inferior Alveolar Nerve Injury Diagnostic and Information System
NR	Not reported
OIDP	Oral Impacts on Daily Performances
PCC	Population–Concept–Context
PRISMA-ScR	Preferred Reporting Items for Systematic Reviews and Meta-Analyses Extension for Scoping Reviews
QST	Quantitative sensory testing
VAS	Visual analogue scale
